# Supplementary figures and images for: Gut Microbiota, Intestinal Morphometric Characteristics, and Gene Expression in Relation to the Growth Performance of Chickens
Source: Animals (Basel). 2022 Dec 9;12(24):3474. doi: 10.3390/ani12243474 (PMC9774407; doi:10.3390/ani12243474)

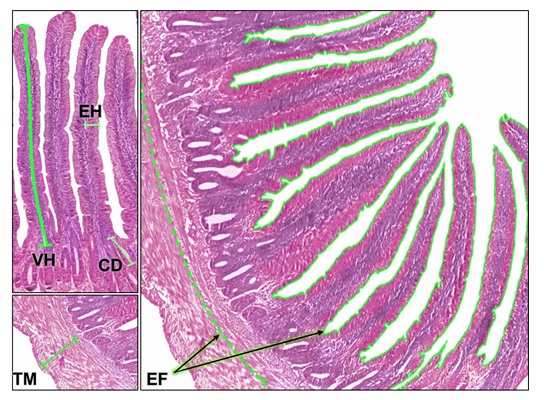

Supplement: Supplementary file 1 [file animals-12-03474-s001.zip › Supplementary Files, Figure S1.jpg]

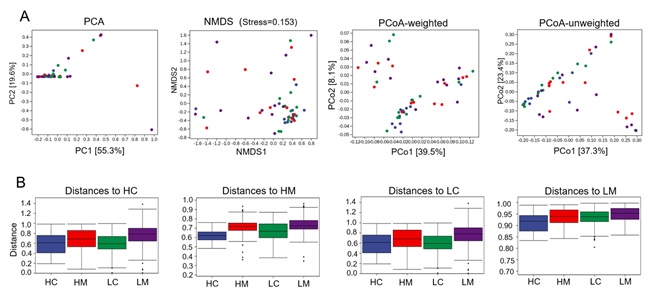

Supplement: Supplementary file 1 [file animals-12-03474-s001.zip › Supplementary Files, Figure S2.jpg]

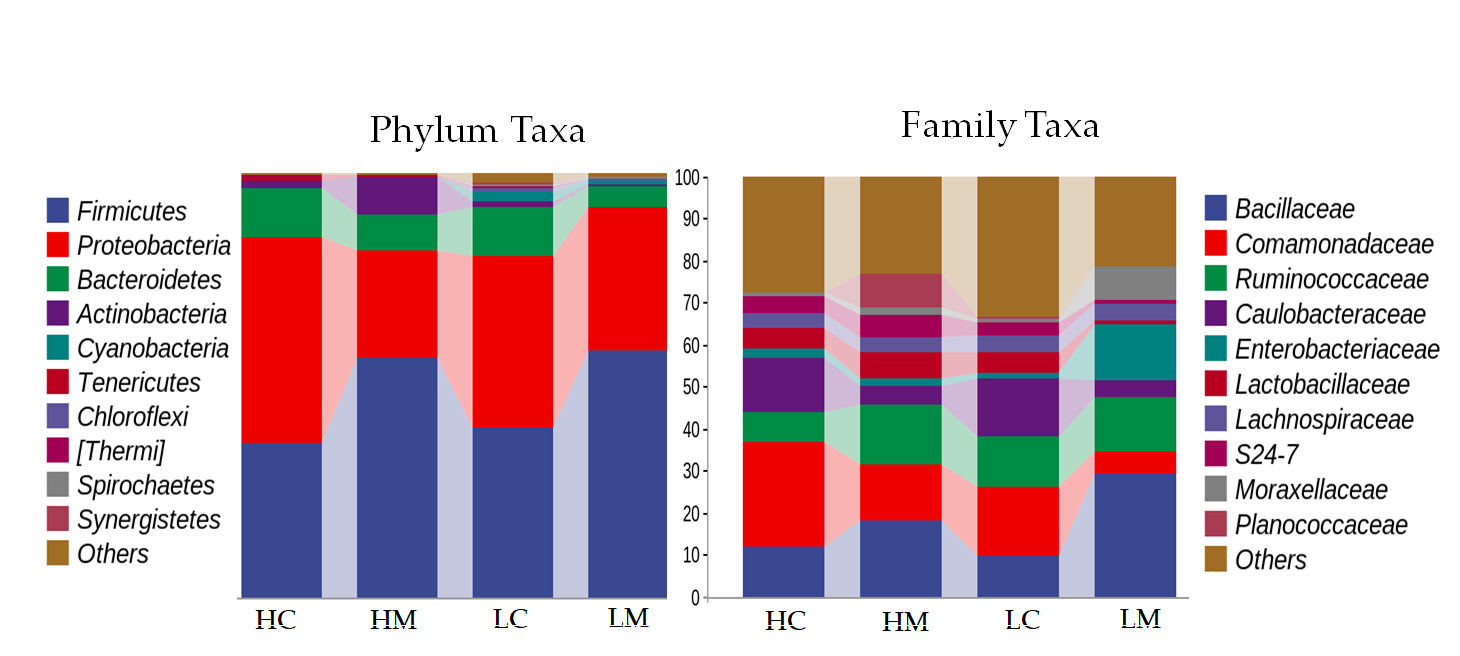

Supplement: Supplementary file 1 [file animals-12-03474-s001.zip › Supplementary Files, Figure S3.jpg]
